# Saving lives through life-threatening measures? The COVID-19 paradox of infection prevention in long-term care facilities

**DOI:** 10.1186/s11556-021-00265-x

**Published:** 2021-06-21

**Authors:** Ansgar Thiel, Dorothee Altmeier, Annika Frahsa, Gerhard W. Eschweiler, Andreas Nieß, Gorden Sudeck, on behalf of the BaSAlt study team

**Affiliations:** 1 Institute of Sport Science, Eberhard Karls University Tübingen, Tübingen, Germany; 2 Interfaculty Research Institute for Sport and Physical Activity, Eberhard Karls University Tübingen, Tübingen, Germany; 3 Institute of Social and Preventive Medicine, University of Bern, Bern, Switzerland; 4 Centre for Geriatric Medicine, University Hospital Tübingen, Tübingen, Germany; 5 Department of Sports Medicine, University Hospital Tübingen, Tübingen, Germany

**Keywords:** COVID-19, Infection prevention, Nursing homes, Long-term care, Physical inactivity

## Abstract

The current SARS Cov-2 infection control measures have paradoxical effects. On the one hand, the lockdown measures help to protect vulnerable populations in particular. On the other hand, these measures inevitably have the effect that those who are to be protected not only become socially isolated and are exposed to enormous psychological stress, but also break down physically due to inactivity. Thus, the activation that is omitted in the lockdown is not compensated by external reference groups, which also indicates that important conditions for healthy ageing are not given in long-term care facilities.

## Summary

The current SARS-Cov-2-infection control measures have paradoxical implications. On the one hand, lockdown measures contribute to the protection of vulnerable populations in particular. On the other hand, the inevitable result of these measures is that those being protected become not only socially isolated and subject to enormous psychological strain, but also decline physically due to prolonged sedentariness.

## Background

Current studies related to the COVID-19 pandemic show that the indisputably necessary measures to restrict contacts have the serious side effect of decreasing physical activity in the population [1]. However, in the public discussion concerning the consequences of the pandemic, the fact that physical activity is one of the most relevant factors in primary, secondary and tertiary prevention of chronic degenerative diseases is rarely addressed. Physical activity is particularly relevant for residents of long-term care facilities due to its preventive effects regarding cardiovascular diseases, the development of sarcopenia and the risk of (neuro-) degenerative diseases [2]. Furthermore, a lack of physical activity is associated with declining cognitive flexibility, gait quality and performance, and depression [3, 4]. In addition, physical activity has positive effects on self-efficacy, mobility, provides opportunities for social interaction, improves well-being, helps maintain quality of life and can help decrease feelings of loneliness or exclusion [5, 6]. The ability of being physically active can thus be considered an important coping strategy for dealing with psychological stressors induced by a crisis such as the SARS-CoV-2 pandemic.

## The infection prevention paradox in long-term care facilities

Against this background, the current infection control measures have paradoxical implications. On the one hand, lockdown measures contribute to the protection of vulnerable populations in particular. On the other hand, the inevitable result of these measures is that those being protected become not only socially isolated and subject to enormous psychological strain, but also decline physically due to prolonged sedentariness [6].

The lockdown setting reduces physical activity in several ways. Firstly, residents are restricted from leaving facilities during local COVID-19 outbreaks. Secondly, visitors – significant motivators for physical activity – are no longer permitted access. Thirdly, caregivers have less time for the residents’ physical activation due to the additional workloads caused by COVID-19. Fourthly, group activities offered by qualified external trainers and providers are either completely discontinued or held by less qualified internal staff and conducted for a shorter duration with reduced numbers of participants – if at all [7].

From this perspective, the limited mobility and restricted social interactions of vulnerable groups in long-term care facilities come with a high price: while weeks of isolation and reduced interactions with visitors could potentially save lives, the lockdown endangers health and potentially increases the risk of mortality in equal measure.

Given that physical activity promotion in long-term care facilities tends not to be structurally embedded with respect to staffing and fixed time slots, there is no substitution for the consequential lack of activating stakeholders [7]. The lockdown thus highlights two main problems with regard to the overall goal of active and healthy ageing in long-term care facilities:

(1) Long-term care facilities are designed to provide protection for the vulnerable but not to empower residents and promote self-determination and autonomy of the residents. The severe lockdown situation reinforces passive-oriented care rather than activating and recognizing residents as experts of their own well-being.

(2) Although physical activity is essential for promoting health and preventing increasing care needs, most long-term care facilities depend on external service providers and visitors to promote physical activity. If these resources are no longer available, activity also comes to a halt.

## Conclusion

The COVID-19 lockdowns should encourage us to rethink the paradigm of resident care, but also the training of caregivers in long-term care facilities:

- Instead of focusing only on protective care for rather passive residents, resource- and activity-oriented empowerment should be the main goal of resident care in long-term care facilities.
- To reduce caregiver role overload, the use of digital tools such as tablets for video communication and video coaching should be incorporated into standard routines in long-term care facilities.
- Long-term care facilities should establish expert networks providing suitable methods for assessing the different physical activity motives and resident needs and for ensuring the quality of offered programs.

Ultimately, the principle for dealing with residents of long-term care facilities should be: *Make the day as challenging as possible in order to maintain Activities of the Daily Living (ADLs) as long as possible.*

## Data Availability

Not applicable.
